# Comprehensive examination of support needs and mental well-being: a mixed-method study of the Austrian general population in times of crisis

**DOI:** 10.3389/fpubh.2024.1345796

**Published:** 2024-05-15

**Authors:** Carina Dinhof, Elke Humer, Katja Haider, Rafael Rabenstein, Andrea Jesser, Christoph Pieh, Thomas Probst, Afsaneh Gächter

**Affiliations:** ^1^Department for Psychosomatic Medicine and Psychotherapy, University of Continuing Education Krems (Danube University Krems), Krems an der Donau, Austria; ^2^Division of Psychotherapy, Department of Psychology, Paris Lodron University Salzburg, Salzburg, Austria; ^3^Faculty of Psychotherapy Science, Sigmund Freud University Vienna, Vienna, Austria

**Keywords:** mental health, support wishes, Austrian general population, mental well-being, mixed-method study

## Abstract

**Introduction:**

In the recent years, the Austrian general population has faced a confluence of multiple crises. This study investigates the support wishes and mental health parameters of the Austrian general population aiming to comprehending the unmet needs and providing guidance for future psychosocial interventions and research endeavors.

**Methods:**

1,031 participants attended the online survey and one third (*n* = 332) wished for further support to improve mental well-being in April 2022. A total of 280 participants accompanied their support wish with written accounts. Participants’ mental health status was evaluated using the PHQ-9 (depression), GAD-7 (anxiety), ISI (insomnia), PSS-10 (perceived stress), CAGE (alcohol abuse), WHO-5 (well-being), and the SCOFF (eating disorder) questionnaires. Data analysis employed a mixed-methods approach.

**Results:**

The preeminent support wish identified was the need for professional mental support (29.3%), followed by communication (21.6%), other professional support except mental and medical support (13.9%). In line with these findings, participants expressing a support wish experienced increased mental health distress across all assessed parameters.

**Conclusion:**

The findings indicate the presence of a vulnerable population within the Austrian general population, which may benefit from targeted support interventions. Consequently, this study contributes to the identification of unmet support needs among the Austrian populace during times of crisis, facilitating the development and enhancement of precisely tailored intervention strategies.

## 1 Introduction

The spreading of the severe acute respiratory syndrome coronavirus type 2 (SARS-CoV-2) and the outbreak of the pandemic in late 2019/early 2020 resulted in major changes in daily life worldwide (1). At the beginning of March 2020, the Austrian general population experienced a decisive turning point in their daily routine due to governmental restrictions, which continued in multiple waves of lockdowns to avoid the spreading of the virus until the end of 2021 (2, 3). Several studies have already shown the impact on mental well-being (4, 5). Deteriorations in mental health indicators were reported, which are potentially a result of living in isolation (6) including social-distancing (7) and quarantine procedures (8) as well as repeatedly closure of school resulting in distance-learning (9–11). At the beginning of the pandemic home-office presented a challenge for employees, especially when they concurrently supervised their children in distance-learning, lacked enough space at home, and experienced blurred lines between private and business life or regarding work breaks (12–14). Commonly, increasing rates of depression (15–17), loneliness (18, 19), insomnia (16), anxiety (3, 16, 17), perceived stress (17) and changes in physical activity (20) were reported.

In the first half of 2022 the governmental restrictions were lifted, but the aftermath regarding physical and mental health of these limitations on the population still needs investigations as conflicting data about the impact on mental health parameters were published (21). Humer et al. assessed various mental health parameter at two-time points (April 2020 and April 2022) showing increased levels of depression but no significant changes in anxiety, insomnia or high stress levels (22). Besides the impact of the COVID-19 pandemic additional stresses and strains occurred due to the massively increased costs for living (inflation) and the Russia-Ukraine war in the beginning of 2022 (23, 24). Worries about the financial situation are found to be more common in the male population whereas women are more prone to worry about negative expectations about health-related consequences of the pandemic (25). Daily news consumption about the COVID-19 pandemic decreased mental health (26–28), which holds true for the Russian-Ukraine war as recently evaluated by Riad et al. (29) for Czech university students where most participants followed the news at least once per day. Furthermore, feeling concerned about the war was significantly higher in female participants (29). Changes in mental health, financial security, and the physical activity of women during the pandemic in North Carolina were evaluated by Zimmerman et al. (30), revealing that participants reported difficulties in paying expenses but also increased levels of anxiety and depression were reported. A recent study in Germany conducted in March 2022 evaluated the fear of war in the German general population (31). Symptoms of depression and anxiety are positively associated with fear of conventional and nuclear war (31) which was further undermined by a study of Massag et al. (32) focusing on three federal states in Germany comparing two time points, where impairment of mental health was reported. Fear of economic crises and worsening of the personal financial situation as well as climate crises were more pronounced after 6 months of the still ongoing Russian-Ukraine war and women were more prone to fear the military expansion (32). Fear of COVID-19 but also fear of a nuclear war contributes to anxiety levels of adults in Portugal (33). The COVID-19 pandemic as well as other crises resulted in an increased level of uncertainty (34–36).

The transactional theory of stress and coping proposed by Lazarus and Folkman (37) emphasizes the dynamic interplay between the individual and their environment. On the one hand, it focuses on primary and secondary cognitive appraisal, positing that stress arises from the subjective evaluation of a situation by the individual (37). On the other hand, coping strategies categorizable into problem-focused coping strategies and emotion-focused coping strategies emerged (37–39). Increased levels of intolerance of uncertainty predict higher levels of anxiety (40) and those individuals are more likely to use maladaptive emotion-focused coping strategies like rumination (35, 36, 41). Social support, humor, optimism, positive thinking and reframing, acceptance, and purpose in life are reported to be beneficial in stressful situation (39). Social support is a multidimensional concept and can be distinguished either into the categories of social ties (friends, family, colleagues, neighbors, etc.) and/or distinct functions including emotional, instrumental/tangible, informational, companionate/social network and esteem support (42–44). Focusing on the role of social support during the COVID-19 pandemic, perceived social support showed a significant inverse correlation with depression, anxiety and stress levels (45–47). Noteworthy, the positive effect of social support from friends and family depends to the quality and quantity (45). Social contacts with friends and families as well as recreational activities are the main areas for support in the Austrian general population as recently shown by Gächter et al. (48). Additionally, that study focused on the main areas of concerns showing that the Austrian populace is primarily concerned about inflation, the Russian-Ukraine war, and mental and physical health (48).

A previous study on wish for support to improve mental health in Austrian school students found that students wishing for professional support showed more often clinically relevant depression, anxiety, and high stress (49). An increased awareness and interest in mental health during the pandemic was reported, showing the wish for a global mental health transformation (50). To extend the results of Gächter et al. (48) and Humer et al. (22), this study examines a new important topic of wish for support to improve mental health in the Austrian general population. More specifically, the following research questions (RQs) were addressed in the study at hand.

RQ 1: Are individuals expressing the wish for support to improve mental health different in sociodemographic and clinical variables from individuals not expressing a wish for this support? (quantitative analysis).

RQ 2: What is the specific kind of support to improve mental health that the individuals wish for? (qualitative analysis).

RQ 3: Are there differences in clinical variables between different categories of support wishes? (quantitative analysis based on qualitative results of RQ 2).

## 2 Materials and methods

### 2.1 Study design and participants

A detailed description of the sampling and recruitment as well as sociodemographic variables of the study population (*N* = 1,031) has already been published by Humer et al. (22). In short: Between 19 and 26 April 2022 an online survey (in German) was conducted in Austria. For participating in this online survey, participants must have been at least 14 years old, must have had an Austrian residency as well as access to the internet and sufficient German language skills. Marketagent.com online research GmbH located in Baden (Austria, certified under ISO 20252) recruited a representative sample of the Austrian population based on pre-existing online access panels. For Austria, about 130,000 registered panelists are available at Marketagent.com. For this study quota sampling was used. Thus, respondents were selected and invited upon following quotas: age, sex, age × sex, region, and educational level. In the supplementary section of Humer et al. (22) a detailed table of the used categories for quota sampling is provided. This table gives insights in the intended quota, based on data from the Austrian Federal Statistics Office compared to the quotas reached for this survey. Following the Declaration of Helsinki and approval of the Ethics Committee of the University of Continuing Education Krems, Austria (Ethical number: EK GZ 26/2018–2021) this study was conducted.

From this study population (*N* = 1,031) the present study evaluated participants expressing a wish for support (*n* = 332) compared to the no-wish-for-support group (*n* = 699) in a mixed methods approach. This study was based on a convergent data transformation design by quantifying some of the qualitative results (51). Out of the wish for support (*n* = 332) a total of *n* = 280 participants further describe their support wish.

### 2.2 Measures

#### 2.2.1 Wish for support (dichotomous question)

The dichotomous question “*Would you like to receive support to improve your mental health*” using yes/no as the answer was used to assess the wish for support in this online survey.

#### 2.2.2 Type of support (open question)

If participants answered the above-mentioned questions with “yes” the subsequent question occurred “*Please describe which kind of support would be helpful for you*.” This question was an open-ended one, thus, participants were able to give a detailed impressions on what type of support they would need. This open-ended question was not only analyzed qualitatively using content analysis but the categories that emerged from the qualitative analysis were additionally used in the subsequent quantitative and mixed-method analyses. Qualitative categories containing more than 30 answers were used to examine differences in the types of support that the Austrian population wished for in relation to their mental health measures.

#### 2.2.3 Sociodemographic characteristics

To assess certain characteristics of the study population following variables were evaluated: gender (male, female, divers); age; educational attainment level (no education, secondary school, apprenticeship, vocational secondary school without general qualification for university entrance, university, higher secondary school including general qualification for university entrance, university); main residence (Vienna, Lower Austria, Upper Austria, Styria, Tyrol, Vorarlberg, Burgenland, Carinthia, Salzburg); monthly net household income (<€ 1,000; € 1,001–€ 2000; € 2001–€ 3,000; € 3,001–€ 4,000; >€ 4,000); migration status (whether participants and/or one or both parents were born abroad); marital status (single or in a relationship).

#### 2.2.4 Well-being index (WHO-5)

The well-being index, developed by the World Health Organization, was used to assess the well-being of individuals (52). The questionnaire contains five items using a six-point scale from 0 to 5. To obtain a percentage scale, the score can be multiplied by four. Thus, it can vary between 0 and 100. A cut-off score of ≤50 is used as an indicator of poor well-being with clinically relevant depressive symptoms. Cronbach’s alpha was 0.904 in the study population.

#### 2.2.5 Patient health questionnaire (PHQ-9)

This questionnaire is composed of nine self-rating items on a four-point scale from 0 to 3 (53). The total score can range from 0 to 27 whereas a higher score indicates more severe depressive symptoms. The PHQ-9 was used to evaluate the depressive symptoms of the study population. A cut-off score of ≥10 defines moderate (i.e., clinically relevant) depressive symptoms. Cronbach’s alpha was 0.89 in the present sample (22).

#### 2.2.6 General anxiety disorder questionnaire (GAD-7)

The GAD-7 questionnaire was used to assess anxiety symptoms (54). This questionnaire consists of seven self-rating items on a four-point scale (0–3). A cut-off score of ≥10 defines moderate anxiety symptoms. Cronbach’s alpha was 0.91 in the study population of *N* = 1,031 (22).

#### 2.2.7 Insomnia sleep index (ISI)

To assess sleep quality and insomnia the five-point scale (from 0 to 4) Insomnia Sleep Index (ISI) was used (55). A cut-off off ≥15 defines clinically relevant moderate insomnia symptoms. Cronbach’s alpha was 0.89 in the study population of *N* = 1,031 (22).

#### 2.2.8 Perceived stress scale (PSS-10)

Levels of perceived stress were measured using the 10 item Perceived Stress Scale (56). Stress levels are measured using a five-point scale from 0 to 4. A cut-off score of ≥14 defines a moderate stress level. Cronbach’s alpha was 0.85 in the study population of *N* = 1,031 (22).

#### 2.2.9 Alcohol abuse (CAGE)

CAGE stands for “Cut down, Annoyance, Guilt, Eye-opener” and is composed of four questions (yes/no) aiming to identify signs of alcoholism (57). A cut-off of two questions answered with “yes” indicates problematic usage of alcohol (58). Cronbach’s alpha was 0.67 in the study population of *N* = 1,031 (22).

#### 2.2.10 Eating disorders (SCOFF)

For screening for eating disorders the Sick, Control, One stone, Fat, Food (SCOFF) questionnaire was used (59, 60). It contains five dichotomous (yes/no) key questions. Two questions answered with “yes” indicate the cut-off value for signs of disordered eating. Cronbach’s alpha was 0.53 in the study population of *N* = 1,031 (22).

### 2.3 Data analysis

#### 2.3.1 Quantitative and mixed method analysis

Descriptive statistics were used to characterize the sociodemographic features of the wish for support and no wish for support group. The mental health parameters, evaluated with the questionnaires WHO-5, PHQ-9, GAD-7, ISI, CAGE, SCOFF, and PSS-10, were compared with between the two “support wish” group (yes/no) in the general population using the Chi-square test. Following a mixed-methods approach, differences in the types of support wishes with respect to mental health parameters were analyzed. *p*-values ≤0.05 were considered as statistically significant. Cramer’s V coefficient (between 0 and + 1) in the Chi-square test indicates the degree of association between two binary variables. Parametric testing was performed using unpaired t-tests. Total scores of the PHQ-9, GAD-7, PSS-10, ISI, and WHO-5 were tested for normal distribution (Supplementary Table 1) using the Kolmogorov-Smirnoff test. Non-parametric testing comparing the wish for support groups was performed using the Mann–Whitney-U test. *p*-values ≤0.05 were considered as statistically significant. For the statistical analyses IBM SPSS Statistics version 29.0.0.0 (IBM Corp., Armonk, New York, United States) was used.

#### 2.3.2 Qualitative data analysis

Responses to the open questions were analyzed using the qualitative data analysis tool ATLAS.ti version 23.1.1.0 (ATLAS-ti scientific software development GmbH, Berlin, Germany). The support wishes varied in their level of detail, since some participants expressed their wish in single keywords while others used whole sentences. The majority only expressed one distinct wish; a minor part (*n* = 38) indicated several support wishes. Coders (C.D and A.G) read through the answers to familiarize themselves with the answers and to get a feeling for the types of responses. This initial reading process was conducted independently from each other. Together both coders developed an initial coding system and did the coding process together. Inconsistencies were discussed and led to a clarification of the categories. Thus, after the first coding phase the category system was revised. The revision of the categories led to distinguishable categories and subcategories with only a few answers (less than five) subsumed in the main categories.

## 3 Results

### 3.1 Sample and study sample characteristics

A total of 1,031 participants (22) attended the survey and 332 participants (33.2%) answered the question “Would you like to receive support to improve your mental health?” with “yes.” The “no support wish” group contains 699 individuals (67.8%). Out of the sample of participants stating a wish for support, 52 participants did not elaborate on their answer to the follow-up question “Please describe which kind of support would be helpful for you.” In more detail, within these 52 participants 45 participants provided no answer by leaving the field empty or entering some random letters or punctuation and 7 answered with “I do not know.” Thus, out of the “support wish” group (*n* = 332) a total of 280 participants described their wishes in more detail.

Table 1 provides a detailed description of the sociodemographic characteristics and difference between the “support wish” (*n* = 332) and the “no support wish” group (*n* = 699). The average age of the participants (*N* = 1,031) was 45.58 ± 17.229 years with 49.7% (*n* = 512) female and 50.3% (*n* = 519) male (22). None of the individuals identified as divers. Within the “support wish” group the mean age was 41.48 years (SD ± 16.476 years) ranging from 14 (*n* = 1) to 83 (*n* = 1) years whereas in the “no support wish” group the average age was 47.53 years (SD ± 17.249). The “support wish” group had a higher proportion of participants aged 25–34 (20.8%) and 35–44 (19.6%) compared to the “no support wish” group which displayed a higher percentage of older participants (range 55–64; 20.3% and above 65, 19.5%). Considerably, in the “support wish” group a higher percentage identified as female (60.8%, *n* = 202) compared to the “no support wish” group, where 45.4% were female and 54.6% were male participants. Furthermore, the difference between those two groups became evident in terms of the monthly net household income. In the “support wish” group the majority (55.8%) had an income below € 2000, while in the “no support wish” group the proportion within this low-income category was about one third (34.5%). Focusing on marital status, 39.8% of the individuals having a wish for support were single compared to the individuals without a support wish (27.2%). Another significant difference was found in employment status where the unemployment rate was higher in the support wish group (23.2%) compared to the no support wish group (17.5%). Participants stating to be retired are more commonly found in the “no support wish” group (26.5%).

**Table 1:** Study sample characteristics of the wish for support group (*N* = 332) and the no wish for support group (*N* = 669).

	Support Wish	No Support Wish	Statistics
	% (N)	% (N)	
Gender			
Female	60.8 (202)	45.4 (317)	ꭓ^2^(1) = 21.612 *p* = < 0.001
Male	39.2 (130)	54.6 (382)	
Age			
14–24	18.7 (62)	11.2 (78)	ꭓ^2^(5) = 29.984 *p* = < 0.001
25–34	20.8 (69)	15.3 (107)	
35–44	19.6 (65)	16.7 (117)	
45–54	15.7 (52)	17.0 (119)	
55–64	15.4 (51)	20.3 (142)	
> 65	9.9 (33)	19.5 (136)	
Age in years (average, ± SD)	41.48 (SD ± 16.476)	47.53 years (SD ± 17.249)	t (1029) = −5.330; *p* = < 0.001
Region			
Vienna	24.7 (82)	20.5 (143)	ꭓ^2^(8) = 4.957 *p* = < 0.762
Upper Austria	16.3 (54)	16.3 (114)	
Lower Austria	17.5 (58)	19.6 (137)	
Carinthia	5.4 (18)	6.2 (43)	
Styria	15.7 (52)	14.0 (98)	
Tyrol	8.1 (27)	8.9 (62)	
Salzburg	5.4 (18)	6.7 (47)	
Burgenland	3.6 (12)	3.1 (22)	
Vorarlberg	3.3 (11)	4.7 (33)	
Education Attainment level			
No education	0.6 (2)	1.0 (7)	ꭓ^2^(5) = 4.783 *p* = < 0.443
Secondary school	23.2 (77)	19.3 (135)	
Apprenticeship	34.3 (114)	33.8 (236)	
Vocational Secondary school (without qualification for university entrance)	17.2 (57)	16.0 (112)	
Higher secondary school (including qualification for university entrance)	15.1 (50)	17.0 (119)	
University	9.6 (32)	12.9 (90)	
Monthly net household income			
< € 1,000	18.1 (60)	10.6 (74)	ꭓ^2^(4) = 47.482 *p* = < 0.001
€1,000–€ 2000	37.7 (125)	23.9 (167)	
€ 2001–€ 3,000	21.4 (71)	26.2 (183)	
€ 3,001–€ 4,000	13.9 (46)	19.0 (133)	
> € 4,000	9.0 (30)	20.3 (142)	
Migration status			
Yes	14.5 (48)	13.2 (92)	ꭓ^2^(1) = 0.332 *p* = < 0.57
No	85.5 (284)	86.8 (607)	
Marital status			
Single	39.8 (132)	27.2 (190)	ꭓ^2^(1) = 16.579 *p* = < 0.001
In a Relationship	60.2 (200)	72.8 (509)	
Employment status			
Employed	59.9 (199)	56.1 (392)	ꭓ^2^(2) = 13.299 *p* = 0.001
Unemployed	23.2 (77)	17.5 (122)	
Retired	16.9 (56)	26.5 (185)	

Migration background was defined with “yes” if participants were born abroad or second-generation immigrants. The *p*-value is given as a two-tailed value.

### 3.2 Mental health parameters and their wish for support

Participants with support wishes have an increased mean score in depression, anxiety, insomnia, high stress, and decreased score in well-being as depicted in Table 2. Especially in terms of depression and anxiety the mean values were twice as high as compared to the “no support wish” group (*n* = 699). Individuals expressing a wish for support (*n* = 332) experienced significantly increased mental health stresses (Mann–Whitney-U, *p* < 0.001) with moderate effect size except for the well-being (weak effect). The questionnaires for alcohol abuse (CAGE) and eating disorders (SCOFF) were excluded from this analysis as there were no mean values calculated due to the design of those questionnaires.

**Table 2:** Mental health parameters with regards to a wish for support.

		Descriptive statistics			Mann–Whitney-U				
	Wish for support	Mean value	SD^1^	SEM^2^	Mean Rank	U-value	Z-value	*p*-value	*r*
Depression (PHQ-9)	Yes	10.65	5.6	0.31	732.15	47259.500	−15.430	< 0.001	0.48
	No	5.00	4.63	0.18	417.61				
Anxiety (GAD-7)	Yes	8.56	4.87	0.28	727.02	45974.000	−15.753	< 0.001	0.49
	No	3.69	3.84	0.16	415.77				
Insomnia (ISI)	Yes	11.49	6.04	0.33	683.48	60432.000	−12.466	< 0.001	0.39
	No	6.55	5.31	0.20	436.45				
Stress (PSS-10)	Yes	20.80	6.07	0.33	717.91	49000.500	−15.020	< 0.001	0.47
	No	13.98	6.42	0.24	420.10				
Well-being (WHO-5)	Yes	45.24	23.19	1.27	391.56	74719.500	−9.262	< 0.001	0.29
	No	59.90	22.82	0.86	575.11				

Wish for support (yes, *n* = 332; no, *n* = 699). The mean value indicates the obtained score in the questionnaires (PHQ-9, GAD-7, ISI, PSS-10, WHO-5) The p-value is given as an asymptomatic, two-tailed value. Effect size is given as r (≤ 0.1–< 0.3, weak; ≤ 0.3–< 0.5, moderate; ≤ 0.5, strong). A decreased value in the WHO-5 (0–50 indicates for poor well-being) indicates a decreased well-being.

1 Standard deviation (SD).

2 Standard error of mean (SEM).

As shown in Table 2, there were significant differences in the mean values between the wish for support groups (yes/no) with the wish for support group showing worse mental health than the no wish for support group. Chi^2^ analyses were performed to investigate differences between the wish for support and no wish for support group in clinically relevant mental health problems using the above described cut-offs (above/below) and revealed that the seven evaluated mental health parameters differed significantly between the “support wish” and “no support wish” group (Table 3). Cramer’s V values indicate a weak effect size for alcohol abuse to moderate size for depression, anxiety, insomnia, eating disorders, high stress and well-being. Throughout all tested parameters, participants wishing for support significantly exceeded the cut-off values more frequently compared to those claiming no support wish.

**Table 3:** Comparison of participants above the cut-off values for the mental health parameters and their wish for support.

	Support wish (*N* = 332)		No Support Wish (*N* = 699)		Statistics ꭓ^2^, *p*-value, φ_c_
Above cut-off^1^	N	%	N	%	
Depression (PHQ-9)	182	54.8	110	15.7	ꭓ^2^ = 169.360 *p* = < 0.001 φ_c_ + 0.405
Anxiety (GAD-7)	120	36.1	47	6.7	ꭓ^2^ = 143.532 *p* = < 0.001 φ_c_ + 0.373
Insomnia (ISI)	90	27.1	60	8.6	ꭓ^2^ = 62.131 *p* = < 0.001 φ_c_ + 0.245
Alcohol Abuse (CAGE)	93	28.0	92	13.2	ꭓ^2^ = 33.714 *p* = < 0.001 φ_c_ + 0.181
Eating Disorder (SCOFF)	147	44.3	122	17.5	ꭓ^2^ = 83.985 *p* = < 0.001 φ_c_ + 0.285
Stress (PSS-10)	54	16.3	20	2.9	ꭓ^2^ = 60.700 *p* = < 0.001 φ_c_ + 0.243
Well-being (WHO-5)^1^	183	55.1	202	28.9	ꭓ^2^ = 66.148 *p* = < 0.001 φ_c_ + 0.253

Wish for support (yes, *n* = 332; no, *n* = 699). The p-value is given as a two-tailed value. Cramer’s V (φ_c_) indicates the degree of association (0 = no relation to + 1 perfect relationship). Cut-off values were determined as follows: PHQ-9 and GAD-7 ≥ 10, ISI ≥ 15, PSS-10 ≥ 14, CAGE and SCOFF with more than 2 answers with “yes,” WHO-5 ≤ 50 (0–50 indicates for poor well-being).

1 Except for WHO-5 where scores is below cut off indicate a poor well-being.

### 3.3 Specified support wishes

A total of 280 participants out of the “support wish” group (*n* = 332) specified their wish for support in the open-ended question. 38 participants expressed more than one wish for support in their answer, thus a total of *N* = 324 responses were subsumed in the category system (Table 4). The qualitative data analysis resulted in 11 main categories and 9 sub-categories, which are shown in Table 4.

**Table 4:** The category system that emerged from the responses to the “Please describe which kind of support would be helpful for you” from the 280 participants providing a free text answer to the open-ended questions on support wishes.

Categories	*N*	% of responses (*n* = 324) of participants providing an answer	% of participants providing an answer (*n* = 280)
Mental support (professional)	95	29.3	33.9
Psychotherapy	66	20.4	23.6
Psychological support	29	9.0	10.4
Communication	70	21.6	25
Conversation	65	20.1	23.2
Social contacts	5	1.5	1.8
Other professional help *(except mental and medical support wishes)*	45	13.9	16.1
Coaching	37	11.4	13.2
Domestic help	4	1.2	1.4
Nutrition counseling	4	1.2	1.4
Medical support	30	9.3	10.7
Wishful thinking	23	7.1	8.2
Recreational activities	19	5.9	6.8
Leisure and Relaxation	17	5.2	6.7
Distraction	2	0.6	0.7
Family support	12	3.7	4.3
Others/not specified	11	3.4	3.9
Financial support	7	2.2	2.5
Self-care	7	2.2	2.5
Work-related wishes	5	1.5	1.8
**Total**	**324**	**100.0%**	**115.7%**

38 participants expressed two wishes of support. Out of those 38 six participants additionally expressed three different types of support wishes. Thus, calculated percentage of participants providing an answer exceed 100%. A total of 280 participants gave 324 responses.

#### 3.3.1 Mental support (professional)

Two years into the COVID-19 pandemic, the most common wish to support people’s well-being was mental support, mentioned by *n* = 95 (33.9%) respondents. The largest subcategory, with *n* = 66 (23.6%), was ‘psychotherapy’, as expressed by Respondent (R) 41: “*A talk with a therapist*.” Some participants also named specific problems that they thought psychotherapy should address, such as R 182: “*Work on my family problems.”* Another subcategory was ‘psychological support’, desired by *n* = 29 (10.4%) participants. Some participants described in detail how they could organize help for themselves, as mentioned by R 145, “*I would contact the professional association of Austrian psychologists to help me with the kind of support.”*

#### 3.3.2 Communication

The second-largest main category in the kind of support, reported by *n* = 70 (25%), was communication. ‘Conversation’ was the first sub-category with *n* = 65 (23.2%) citations. The participants wished to have some kind of exchange, to be able to communicate or to confide their problems or concerns to someone, as described by R 36, “*That I can confide my concerns to someone who does not blurt everything out and to be able to tell a friend everything that worries me without being deceived.*” For some participants, it seemed to be important to have someone to talk to, as R 53 explained: “*Just someone to listen would maybe make a difference*.” Other participants considered whether talking to similar-minded people would help them, as noted by R 136: “*I think that I could talk about my problems with like-minded people; this might be helpful.*” ‘Social contacts’ as a second sub-category were mentioned by *n* = 5 (1.8%) participants, such as R 58: “*Just meeting up with old acquaintances again.”*

#### 3.3.3 Other professional help (except mental and medical support wishes)

The third-largest category for improving mental well-being encompassed different types of professional support (mental and medical support wishes excluded), which were identified by *n* = 45 (16.1%) participants. This category was composed of the following subcategories: The largest sub-category with *n* = 37 (13.2%) was the desire for coaching such as accompaniment, individual support, relaxation therapy, or counseling in the case of financial debts. Some of the participants wished for counseling for all areas of their lives, as expressed by R 165, “*Life counsellor for all areas, be it nutrition, time management, exercise, finances*.” Or professional help to achieve something, as R 140 mentioned, “*I would like a personal trainer.*” The second subcategory was domestic help, which was mentioned by *n* = 4 (1.4%) participants, who could no longer manage their household or want “*someone to be there and take care of them”* (R 148). Furthermore, *n* = 4 (1.4%) reported that they would like nutrition counseling to “*learn proper eating behavior.”* (R 61).

#### 3.3.4 Medical support

This main category consisted of different wishes for medical support, which were reported by *n* = 30 (10.7%). In addition to medical help or control, special forms of treatment such as health treatment or rehabilitation were also expressed. Some participants wanted medical help for pain management, such as R 32 expressed, “*Need someone to help me with my pain.”* In addition, participants wished for medication, psychopharmacological, psychiatric support or specifically *“hospitalization in a psychiatric clinic”* (R 63).

#### 3.3.5 Wishful thinking

The category wishful thinking with *n* = 22 (8.2%) consisted of answers that can be described as wish-led thoughts that should lead to mental well-being for the respondents. The wishes varied, such as the removal of the government’s measures to contain the COVID-19 pandemic or to “*take time off without money worries”* (R 21). Some wishes were related to partners, such as “*getting more privacy from the partner”* (R 83) or wishing for a partnership or intimacy.

#### 3.3.6 Recreational activities

This main category, recreational activities, reported by *n* = 19 (6.8%), refers to both outdoor and indoor activities and consists of two subcategories. ‘Leisure and relaxation’ as a wish for support was reported by *n* = 17 (6.1%) respondents. More outside movement was noted by R 179, “*More time in the fresh air and less stress*.” To increase well-being, certain techniques are necessary, as R 286 pointed out, “*to generally increase well-being, be it breathing exercises or sports,* etc.*”* Other recreational activities mentioned were taking a holiday to relax, creating leisure time, or reading books. The second subcategory was distraction, described by *n* = 2 (0.7%).

#### 3.3.7 Further main categories

A further main category that could contribute to the improvement of mental well-being was the wish for family support, which was described by *n* = 12 (4.3%) respondents. The main category Others/not specified, reported by *N* = 11 (3.9%), was selected when the respondents gave no specific input regarding what could be done to improve mental well-being (R 161 expressed: “offers free of charge”). Financial support was stated by *n* = 7 (2.5%) participants as being useful, and some respondents (*n* = 7, 2.5%) stated that their mental well-being could be improved if they took care of themselves and took the time or for example R 30 noted: “*Sleep better and longer.”* The last main category concerned work-related wishes (*n* = 5, 1.8%) and referred to reduced working hours to improve well-being or as R 282 expressed, “*Reduced working hours or workload by additional staff*.”

### 3.4 Support wishes and the mental health parameters

Chi^2^ tests were performed to investigate differences in clinically relevant mental health problems between the wish for support and no wish for support group within each of the three main wish for support categories (mental support, communication, professional support; yes/no). The calculated Chi^2^ tests (Supplementary Tables 2–4) showed that the seven evaluated mental health parameters did not differ significantly between the wish for support and no wish for support group within these three specific wish for support categories (mental support, communication, professional support).

## 4 Discussion

The primary objective of this study was to investigate the support wishes of the Austrian general population in times of multiple crises (Russia-Ukraine war, inflation, and the COVID-19 pandemic era). This examination was conducted as a mixed method approach based on insights of from previously published works from Humer et al. (22) and Gächter et al. (48). During the time of data collection in April 2022 the governmental restrictions preventing the spreading of the SARS-CoV-2 virus were lifted. However, the aftermath of those strict limitations and additional stresses and strains due to the inflation and war affected the Austrian general population.

A comparison of the sociodemographic variables of the “support wish” and “no support wish” group revealed significant differences in terms of gender, age, monthly net household income, martial and employment status. The support wish group comprises more female as well as younger-aged participants and those with lower income and/or unemployed individuals. Earlier studies already established links between sociodemographic factors and mental health parameters (22, 61–63). Employment was identified as a factor that fosters self-efficacy and a sense of purpose, thereby promoting resilience in the face of impending adversities and crises (64, 65).

Individuals wishing for mental health support in this presented study had distinct higher scores across several mental health parameters evaluating depression (PHQ-9), insomnia (ISI), anxiety (GAD-7), high stress (PSS-10), and lower well-being (WHO-5). Of particular interest, the mean scores of the above-mentioned questionnaires were consistently about twice as high in the “support wish” group as in the “no support wish” group and exceeded the cut-off values for depression and high stress. Strikingly, it became evident that individuals expressing a support wish exceeded the cut-off values more frequently in all tested parameters compared to the “no support wish” group. More than half of the support wish group (54.8% PHQ-9; 55.1% WHO-5) showed a clinically relevant score in depression, followed by 44.3% of participants expressing symptoms of an eating disorder. Around one-third of individuals in the “support wish” group showed signs of anxiety (36.1%), insomnia (27.1%) and alcohol abuse (28.0%). These findings allude to the presence of a vulnerable group within the general population, where additional interventions could treat and deter the development of clinically relevant mental illnesses.

Noteworthy, our surveyed population is aware of mental health issues, which is in line with recent findings of the World Health Organization (WHO) (50). More than one-third of respondents (33.9%) expressing a desire for additional support referred to mental health problems. This indicates an existing acceptance of and demand for psychological or psychotherapeutic treatment options. Furthermore, it involves efforts to directly cope with or change the stressor. Problem-orientated coping strategies, as described by Lazarus and Folkman, aim to change, reduce or eliminate stressful situations and their impact (37). Problem-solving approaches by seeking professional help to bring about a change in one’s own environment or behavior would be one example for this above-mentioned coping strategy.

However, it is conceivable that available services remain underutilized due to a lack of awareness or mismatch with the needs of this subgroup. Notably, a study among Austrian school students revealed that their primary support wish was professional help (49). In Austria the costs for psychotherapy are often only partly covered by health insurances. Cost-reimbursements are possible, if the psychotherapist is already registered at the Federal Ministry of Social Affairs, Health, Care and Consumer Protection (66). Considering the concerns about inflation and increased costs of living (48) as well as the low monthly net household income, individuals may hesitate to seek professional mental health support. This hesitancy could pose a risk factor for Austria’s healthcare, social, and pension systems, as mental health conditions have been linked to high rates of sick leave and early retirements (67). A recent study identifies “having a job” as one protective factor for general psychiatric disorders (68). Regarding this finding, work stress and work–family conflicts correlate with affective disorders, which can be prevented with work-family initiatives including flexible work time and place (69). Therefore, it is imperative to enhance the availability of support services or create offerings tailored to the needs of individuals, enabling them to navigate through times of uncertainty and distress while maintaining their capacity to work.

Not only did COVID-19 increase the economic uncertainty (70), but additionally the Russian-Ukraine affected the food and gas supply chains in Europe resulting in increased prices (71, 72). Extensive media coverage of both, the inflation and the Russian-Ukraine war, may have left the population feeling insecure. Gächter et al. (48) demonstrated that in April 2022, the primary concerns of the Austrian general population centered on inflation, challenges in covering monthly expenses, worries related to the Russian-Ukraine war, and mental health. Feelings of overwhelm and uncertainty are known drivers of mental health distress (41).

Noteworthy findings were observed in the main category of support wishes summed up under the term communication. Participants expressed a desire to communicate or share their concerns with someone and wished for companionship in times of distress. These findings align with similar results obtained by Haider et al. (49) where Austrian students expressed a desire to talk to someone without specialized expertise, including friends and family. Social contacts and support play a pivotal role during times of stress, serving as essential resources contributing to resilience (73). A qualitative online survey conducted by Schaffler et al. (38) in December 2020 revealed that, especially in the initial year of the COVID-19 pandemic, social contacts were the primary source of support. While the present dataset also emphasized the importance of social contacts as a main source of support (48), it is noteworthy that the percentage of individuals relying on social contacts declined from 46% in 2020/21 to 36.2% in 2022. Changes and challenges in friendships were evaluated by Kulcar et al. (73) highlighting the difficulty of forming new acquaintances during the COVID-19 pandemic and a decrease in the number of relationships as the pandemic persisted. Consequently, building and maintaining friendships were negatively affected during this period (73). Schaffler et al. (38) found that social support as well as recreational activities and distractions, played a crucial role. These findings underscore the susceptibility of the population to the crises of 2022 and emphasize the central role of communication and support to promote individuals’ coping mechanisms. Our research revealed that social support, primarily through communication channels such as conversation and social interactions, emerged as the second most sought-after form of support. Within this category, participants expressed a desire for empathy, understanding, and interaction with like-minded individuals. This aligns with previous research indicating that perceived social support during challenging times, such as the COVID-19 pandemic, correlates with reduced levels of depression, anxiety, and stress (45–47). These observations resonate with Lazarus and Folkman’s assertion that the effectiveness of coping strategies depend on individuals’ appraisal of the stressor and their perceived capacity to manage it (37). Moreover, Lueger-Schuster et al. (3) emphasized the necessity of pandemic-related coping behaviors, including maintaining a healthy lifestyle, adherence to prevention measures, and engaging in joyful activities. Individuals affected by crises and lacking adequate coping strategies may greatly benefit from acquiring and enhancing active coping skills, such as acceptance, positive thinking, and seeking social support (39). These results harmonize with the wishes expressed for mental support, professional assistance, and enhanced communication.

Taking those results together the presented “support wish” group represents a vulnerable group in terms of mental distresses of the Austrian general population. As multiple crises occurred between 2020 and 2022 the mental burden on the population intensified. The Austrian populace was confronted with the COVID-19 pandemic, replete with associated fears, concerns, and containment measures, as well as the financial repercussions of the pandemic, which were further exacerbated by the Russian-Ukraine war and subsequent inflation. This study has spotlighted unmet needs and catalyze discussions and initiatives aimed at improving the availability of mental health services in Austria, along with advocating for further research on this critical topic.

In conclusion, the study findings underscore the urgency of addressing the mental health needs of the Austrian population amidst multiple crises, highlighting the relevance of understanding individuals’ responses to stressors and their coping mechanisms. Proactive measures focusing on the adaption to the needs, effective coping strategies and availability of mental health services are essential for fostering resilience in the face of future uncertainties and crises.

## 5 Limitations

The results of the present study should be beheld under consideration of the following limitations. First, the study was conducted as an online survey, thus no face-to-face interviews were done and only written responses were analyzed. Second, during the time of the online survey, daily news and debates about the Russian-Ukraine war and inflation were present and might have influenced the response behavior. The COVID-19 prevention measures of the government were lifted during this period. Third, no standardized scale for “support wish” was used, responses were collected in an open-ended-format giving the chance to explore the answers. Fourth, it was not quantitatively evaluated, if the participants already received any kind of professional support. Therefore, participants might be above the cut-off values and already receiving support and not expressing additional wishes for further help, especially in mental health matters. Fifth, although in the frame of this online survey a representative sample of the Austrian general population was recruited, only a small group expressed a wish for support. Given the variety of possible answers the sample size in some categories was too small for statistical analyses and the non-significant results for RQ 3 (differences in mental health indicators between wish for support and no wish for support groups within specific wish for support subgroups) might also be attributable to the smaller sample sizes in these “wish for specific support” subgroups. Sixth, this study was designed as a cross-sectional study. Tracking participants over a certain time, especially about the changes and duration of crises, would potentially provide a more in-depth analysis of changes in the mental health parameters and wishes for support over time.

## 6 Conclusion

This study aimed to analyze the wish for support to improve mental health in the Austrian general population in times of crises under consideration of the mental health status and the individual self-reported wishes for support in April 2022. Further in-depth analysis of the subpopulation expressing a support wish should be in the scope of follow-up studies. For further research, focusing on this vulnerable subgroup to analyze the obstacles in seeking mental, medical, and professional help would be recommended.

## Data availability statement

The raw data supporting the conclusions of this article will be made available by the authors, without undue reservation.

## Ethics statement

The studies involving humans were approved by Ethics Committee of the University of Continuing Education Krems, Austria (Ethical number: EK GZ 26/2018–2021). The studies were conducted in accordance with the local legislation and institutional requirements. The participants provided their written informed consent to participate in this study.

## Author contributions

CD: Conceptualization, Formal analysis, Investigation, Methodology, Visualization, Writing – original draft. EH: Writing – review & editing, Conceptualization, Data curation, Investigation, Methodology. KH: Writing – review & editing. RR: Writing – review & editing. AJ: Writing – review & editing. CP: Writing – review & editing. TP: Supervision, Writing – review & editing. AG: Conceptualization, Methodology, Supervision, Visualization, Writing – original draft, Writing – review & editing, Formal analysis, Investigation.
