# Supplementary material for: Comprehensive examination of support needs and mental well-being: a mixed-method study of the Austrian general population in times of crisis
Source: Front Public Health. 2024 May 15;12:1345796. doi: 10.3389/fpubh.2024.1345796 (PMC11133677; doi:10.3389/fpubh.2024.1345796)
Supplement: Supplementary file 1 [file Table_1.DOCX]

Supplementary Material

# Supplementary Table

Supplementary Table 1: Testing for normal distribution

|  | **Testing for normal distribution** | |
| --- | --- | --- |
|  | **Kolmogorov-Smirnov** | |
|  | statistics | p-value |
| Depression (PHQ-9) | 0.122 | ***p < 0.001*** |
| Anxiety (GAD-7) | 0.135 | ***p < 0.001*** |
| Insomnia (ISI) | 0.103 | ***p < 0.001*** |
| Stress (PSS-10) | 0.047 | ***p < 0.001*** |
| Well-being (WHO-5) | 0.103 | ***p < 0.001*** |

*Note. The scores of the above-mentioned questionnaires were tested for normal distribution (n=1,031) A p-value below 0.05 is considered as significant leading to the rejection of the null-hypothesis (H0 = data is normal distributed).*

Supplementary Table 2: Proportion of participants exceeding cut-off scores for mental health symptoms within participants expressing a wish for mental support (professional) (n = 94)^1^ compared to those expressing no explicit wish for mental support (n = 186). Only participants responding to the open-ended question on specific support wishes to improve mental well-being (n = 280) are included in the analyses.

|  |  | **Mental (professional) Support Wish** | |  |
| --- | --- | --- | --- | --- |
| **Variable** |  | **Yes  (n = 94)^1^** | **No  (n =186)** | **Statistics** |
| **Depression**  **(PHQ-9)** | % | 58.5 | 51.6 | ꭓ² (1\|280) = 1.196;  p = 0.274; φ_c_ +0.065 |
|  | (n) | (55) | (96) |  |
| **Anxiety**  **(GAD-7)** | % | 41.5 | 32.3 | ꭓ² (1\|280) =2.328;  p = 0.127; φ_c_  +0.091 |
|  | (n) | (39) | (60) |  |
| **Insomnia**  **(ISI)** | % | 26.6 | 27.4 | ꭓ² (1\|280) = 0.021;  p = 0.884; φ_c_  +0.009 |
|  | (n) | (25) | (51) |  |
| **Alcohol Abuse (CAGE)** | % | 27.7 | 28.5 | ꭓ² (1\|280) = 0.21;  p = 0.883; φ_c_  +0.009 |
|  | (n) | (26) | (53) |  |
| **Eating Disorder (SCOFF)** | % | 39.4 | 43.5 | ꭓ² (1\|280) = 0.449;  P = 0.294; φ_c_  +0.040 |
|  | (n) | (37) | (81) |  |
| **Stress**  **(PSS-10)** | % | 19.1 | 16.7 | ꭓ² (1\|280) = 0.266;  p = 0.606; φ_c_  +0.031 |
|  | (n) | (18) | (31) |  |
| **Well-being**  **(WHO-5)** | % | 63.8 | 54.3 | ꭓ² (1\|280) = 2.320;  P = 0.128; φ_c_  +0.091 |
|  | (n) | (60) | (101) |  |

*Note*. Cut-off values were determined as follows: PHQ-9 and GAD-7 ≥ 10, ISI ≥ 15, PSS-10 ≥ 14, CAGE and SCOFF with more than 2 answers with “yes”, WHO-5 ≤ 50 (0-50 indicates poor well-being). The p-value is given as a two-tailed value. Cramer’s V (φ_c_) indicates the degree of association (0 = no relation to +1 = perfect relationship). A p-value below 0.05 is considered as significant.
^1^ One participant expressed two wishes which subsumed into “mental support”. For this analysis, one entry per person was included.

Supplementary Table 3: Proportion of participants exceeding cut-off scores for mental health symptoms within participants expressing a support wish for communication (n = 68)^1^ compared to those expressing no explicit wish for mental support (n = 212). Only participants responding to the open-ended question on specific support wishes to improve mental well-being (n = 280) are included in the analyses.

|  |  | **Communication Support Wish** | |  |
| --- | --- | --- | --- | --- |
| **Variable** |  | **Yes  (n = 68)^1^** | **No  (n = 212)** | **Statistics** |
| **Depression**  **(PHQ-9)** | % | 47.1 | 56.1 | ꭓ² (1\|280) = 1.706  p = 0.192; φ_c_  +0.078 |
|  | (n) | (32) | (119) |  |
| **Anxiety**  **(GAD-7)** | % | 33.8 | 35.8 | ꭓ² (1\|280) = 0.092  p = 0.761; φ_c_  +0.018 |
|  | (n) | (23) | (76) |  |
| **Insomnia**  **(ISI)** | % | 26.5 | 27.4 | ꭓ² (1\|280) = 0.021  p = 0.886; φ_c_  +0.009 |
|  | (n) | (18) | (58) |  |
| **Alcohol Abuse (CAGE)** | % | 20.6 | 30.7 | ꭓ² (1\|280) = 2.579  p = 0.108; φ_c_  +0.096 |
|  | (n) | (14) | (65) |  |
| **Eating Disorder (SCOFF)** | % | 41.2 | 42.5 | ꭓ² (1\|280) = 0.034  p = 0.853; φ_c_  +0.011 |
|  | (n) | (28) | (90) |  |
| **Stress**  **(PSS-10)** | % | 17.6 | 17.5 | ꭓ² (1\|280) = 0.001  p = 0.971; φ_c_  +0.002 |
|  | (n) | (12) | (37) |  |
| **Well-being**  **(WHO-5)** | % | 55.9 | 58.0 | ꭓ² (1\|280) = 0.096  p = 0.756; φ_c_  +0.019 |
|  | (n) | (38) | (123) |  |

*Note*. Communication support wish (n = 68). The p-value is given as a two-tailed value. Cramer’s V (φ_c_) indicates the degree of association (0 = no relation to +1 = perfect relationship). Cut-off values were determined as follows: PHQ-9 and GAD-7 ≥ 10, ISI ≥ 15, PSS-10 ≥ 14, CAGE and SCOFF with more than 2 answers with “yes”, WHO-5 ≤ 50 (0-50 indicates poor well-being). A p-value below 0.05 is considered as significant.

^1^ Two participant expressed two wishes which subsumed into “communication support”. For this analysis, one entry per person was included.

Supplementary Table 4: Proportion of participants exceeding cut-off scores for mental health symptoms within participants expressing a support wish for other professional support except mental and medical support (n = 42)^1^ compared to those expressing no explicit wish for mental support (n = 212). Only participants responding to the open-ended question on specific support wishes to improve mental well-being (n = 280) are included in the analyses.

|  |  | **Other professional support (except mental/medical support) Support Wish** | |  |
| --- | --- | --- | --- | --- |
| **Variable** |  | **Yes  (n = 42)^1^** | **No  (n = 238)** | **Statistics** |
| **Depression**  **(PHQ-9)** | % | 52.4 | 54.2 | ꭓ² (1\|280) = 0.048  p = 0.827; φ_c_  +0.013 |
|  | (n) | (22) | (129) |  |
| **Anxiety**  **(GAD-7)** | % | 35.7 | 35.3 | ꭓ² (1\|280) = 0.003  p = 0.958; φ_c_  +0.003 |
|  | (n) | (15) | (84) |  |
| **Insomnia**  **(ISI)** | % | 26.2 | 27.3 | ꭓ² (1\|280) = 0.023  p = 0.880; φ_c_  +0.009 |
|  | (n) | (11) | (65) |  |
| **Alcohol Abuse (CAGE)** | % | 19.0 | 29.8 | ꭓ² = 2.050  p = 0.152; φ_c_  +-0.086 |
|  | (n) | (8) | (71) |  |
| **Eating Disorder (SCOFF)** | % | 42.9 | 42.0 | ꭓ² (1\|280) = 0.010  p = 0.919; φ_c_  +0.006 |
|  | (n) | (18) | (100) |  |
| **Stress**  **(PSS-10)** | % | 19.0 | 17.2 | ꭓ² (1\|280) = 0.082  p = 0.775; φ_c_  +0.017 |
|  | (n) | (8) | (41) |  |
| **Well-being**  **(WHO-5)** | % | 57.1 | 57.6 | ꭓ² (1\|280) = 0.003  p = 0.959; φ_c_  +-0.003 |
|  | (n) | (24) | (137) |  |

*Note*. Professional support wish (n = 42). The p-value is given as a two-tailed value. Cramer’s V (φ_c_) indicates the degree of association (0 = no relation to +1 = perfect relationship). Cut-off values were determined as follows: PHQ-9 and GAD-7 ≥ 10, ISI ≥ 15, PSS-10 ≥ 14, CAGE and SCOFF with more than 2 answers with “yes”, WHO-5 ≤ 50 (0-50 indicates poor well-being). A p-value below 0.05 is considered as significant.

^1^ Three participant expressed two wishes which subsumed into “communication support”. For this analysis, one entry per person was included.
